# Deciphering Automation Transparency: Do the Benefits of Transparency Differ Based on Whether Decision Recommendations Are Provided?

**DOI:** 10.1177/00187208251318465

**Published:** 2025-02-03

**Authors:** Isabella Gegoff, Monica Tatasciore, Vanessa K. Bowden, Shayne Loft

**Affiliations:** 1 2720 The University of Western Australia, Australia

**Keywords:** automation transparency, decision recommendations, human-automation teaming, uninhabited vehicle management

## Abstract

**Objective:**

To better understand automation transparency, we experimentally isolated the effects of additional information and decision recommendations on decision accuracy, decision time, perceived workload, trust, and system usability.

**Background:**

The benefits of automation transparency are well documented. Previously, however, transparency (in the form of additional information) has been coupled with the provision of decision recommendations, potentially decreasing decision-maker agency and promoting automation bias. It may instead be more beneficial to provide additional information without decision recommendations to inform operators’ unaided decision making.

**Methods:**

Participants selected the optimal uninhabited vehicle (UV) to complete missions. Additional display information and decision recommendations were provided but were not always accurate. The level of additional information (no, medium, high) was manipulated between-subjects, and the provision of recommendations (absent, present) within-subjects.

**Results:**

When decision recommendations were provided, participants made more accurate and faster decisions, and rated the UV system as more usable. However, recommendation provision reduced participants’ ability to discriminate UV system information accuracy. Increased additional information led to faster decisions, lower perceived workload, and higher trust and usability ratings but only significantly improved decision (UV selection) accuracy when recommendations were provided.

**Conclusion:**

Individuals scrutinized additional information more when not provided decision recommendations, potentially indicating a higher expected value of processing that information. However, additional information only improved performance when accompanied by recommendations to support decisions.

**Application:**

It is critical to understand the potential differential impact of, and interaction between, additional display information and decision recommendations to design effective transparent automated systems in the modern workplace.

## Introduction

Modern workplaces increasingly use automated decision aids to provide human operators with information and/or recommendations on task actions (Mosier & Manzey, 2020). Decision aids have improved safety and productivity in work domains such as aviation, healthcare, and transportation (NASEM, 2022). These benefits extend to the modern military battlefield where, for example, decision aids provide recommendations regarding the management of uninhabited vehicles (UVs; U.S. Air Force, 2015). Decision aids are often highly reliable in such contexts but may not be perfect and can provide inaccurate information/recommendations, potentially leading to automation *misuse* (accepting incorrect advice) or *disuse* (rejecting correct advice; Lee & See, 2004; Parasuraman & Riley, 1997).

Automation *transparency* has been suggested as a work design principle to improve the calibration of human trust in automation and appropriate reliance on automated advice. Automation transparency has been conceptualized in various ways. For instance, it has been defined as providing “real-time understanding of the actions of the AI system” (NASEM, 2022, p. 31), thereby increasing the “understandability and predictability of [a] system” (Endsley et al., 2003, p. 146). In line with this, the Situation-Awareness Agent-Based Transparency (SAT; Chen et al., 2014) model outlines three levels of transparency: the automation’s goals and intentions (Level 1), the reasoning behind automated advice (Level 2), and further reasoning regarding the projected outcomes if advice is followed (Level 3). While the SAT model is often referenced, transparency design ultimately varies depending on the task domain in which the automation is utilized (Van de Merwe et al., 2022, 2024).

Research across various task domains shows that increased transparency improves the accuracy of automation use (e.g., Skraaning & Jamieson, 2021), either decreases or at least does not detrimentally impact decision time and perceived workload (e.g., Göritzlehner et al., 2014; Panganiban et al., 2020), and can increase trust in automation and usability ratings (see reviews of transparency effects by Bhaskara et al., 2020; Sargent et al., 2023; Van de Merwe et al., 2022). Transparency can also mitigate the increased disuse of automated advice associated with low-reliability automation (Gegoff et al., 2024). However, transparency can increase bias towards agreeing with automation, either leading to no improvement in correct rejection rates (e.g., Tatasciore et al., 2023; Tatasciore & Loft, 2024), or at times decreased correct rejection rates (Bhaskara et al., 2021; Bussone et al., 2015).

To date, however, transparency manipulations have always been coupled with automated advice (decision recommendations). To our knowledge, no prior studies have attempted to disentangle the benefits of providing increased transparency from the impact of providing the recommendations themselves. We do so in the current study. Henceforth, we use the term increased *additional information* when recommendations are provided to indicate increased transparency. This is because the term *transparency* often refers to the provision of additional information designed to increase understanding of the *rationale* underlying recommendations, and to increase its predictability for individuals to correctly accept/reject decision recommendations. In the current study, we include conditions in which the same additional information was provided with or without accompanying decision recommendations. Without recommendations, individuals could use the additional information to inform their own unaided decisions. For this reason, we used the term additional information instead of transparency, while noting that when additional information *is* coupled with recommendations, additional information is conceptually analogous to transparency.

While we use the term “unaided,” we acknowledge that “additional information” without recommendations may constitute *low degree of automation* (Wickens et al., 2010) support to the extent that it presents an analysis and integration of task-goal relevant information (Parasuraman et al., 2000). An example is air traffic control automation that provides an ordered list of predicted separation between aircraft pairs. This point notwithstanding, the central and novel premise of the current paper, as discussed below, is that the extent to which operators scrutinize additional information, and their subsequent decision accuracy, may depend on whether additional information is presented alongside recommendations or not.

## Does the Impact of Additional Information Depend on Recommendation Provision?

Performance on a task in which decision recommendations are provided depends on the confluence of three factors: the ability of the human, automation reliability, and strategies the human uses to integrate their judgment with recommendations (Bahrami et al., 2010; Strickland et al., 2021, 2023). Unfortunately, multiple inefficiencies have been identified regarding how operators integrate information from raw task inputs with recommendations (Bartlett & McCarley, 2017, 2021; Boskemper et al., 2022; Tikhomirov et al., 2023; Wang et al., 2009), such as operators underweighting recommendations or deferring to them uncritically. These inefficiencies are influenced by operator trust in automated systems (Matthews et al., 2020). Nonetheless, despite falling short of optimal performance, operators typically make more accurate decisions when provided recommendations (Wickens & Dixon, 2007). Therefore, under conditions in which no additional information is presented, we expected participants in the current study to make more accurate decisions when provided recommendations.

It may be critical to provide decision recommendations with additional information to realize the aforementioned benefits (Bhaskara et al., 2020; Sargent et al., 2023; Van de Merwe et al., 2022). However, some researchers contend that providing recommendations, even if coupled with additional information (i.e., transparency), can lead to over-reliance on recommendations because it reduces decision-maker agency (Endsley, 2017; Miller, 2023), a sentiment consistent with findings of increased bias towards agreeing with recommendations when presented additional information (e.g., Bussone et al., 2015; Tatasciore & Loft, 2024). In line with this, Strickland et al.’s (2021, 2023) computational model of automation use identified that humans inhibit decisions disagreeing with recommendations. Operators may not always engage with or scrutinize additional information to the extent required to optimize decision making if also provided recommendations (Miller, 2023). In contrast, when provided the same additional information without recommendations, operators may hold a higher *expected value* of processing that information to make decisions (Moray, 2003; Senders, 1983; Wickens et al., 2015). Endsley (2017) concluded that automation that provides information to enhance situation awareness through improved information integration, but without recommendations, should lead to more accurate automation use.

On this basis, it may be beneficial, or at least not detrimental, to provide additional information without accompanying recommendations. In this case, the benefit of additional information could be greater when no recommendation is provided compared to when provided. However, it is unknown whether this would cost decision time or perceived workload (henceforth referred to as workload) given the increased processing demands on the operator to analyze additional information without accompanying recommendations.

## The Current Study

To our knowledge, no prior study has experimentally isolated the effect of providing additional information from the effect of providing decision recommendations. We examined the effect of additional information when recommendations were provided and not provided on decision accuracy, decision time, workload, trust, and system usability.

The UV management task used in the current study has been previously used in automation transparency research (Gegoff et al., 2024; Tatasciore et al., 2023, 2024; Tatasciore & Loft, 2024) and is particularly relevant to Defense, but also broadly representative of other modern work contexts. Studies examining the impact of transparency (i.e., when additional information was coupled with decision recommendations) in UV tasks have found that increased transparency improves the accuracy of automation use (e.g., Gegoff et al., 2024; Mercado et al., 2016; Stowers et al., 2020; Tatasciore & Loft, 2024; Tatasciore et al., 2023, but see Bhaskara et al., 2021) without costs to, or even benefits to, decision time and workload (Gegoff et al., 2024; Tatasciore et al., 2024). Of course, this does not necessarily mean that optimal performance was reached (Bartlett & McCarley, 2021; Tikhomirov et al., 2023), and these prior studies did not experimentally isolate additional information from recommendations.

In the current study, participants selected the optimal UV to complete missions in a mixed design, with additional information (no, medium, high) manipulated between-subjects and decision recommendation (no recommendation provided, recommendation provided) manipulated within-subjects. The SAT model was used as a guide to design medium (SAT Level 2) and high (SAT Level 3) additional information, but additional information designs were also informed via consultation with subject matter experts at the Australian Defense Science and Technology Group (i.e., observation and discussion of UV management platforms). As discussed earlier, the term *additional information* is used here rather than *transparency* because additional information only qualifies as transparency when coupled with recommendations.

Study predictions are summarized in Table 1. We expected UV selection accuracy to increase with the provision of additional information (present vs. absent), and with increased additional information (high vs. medium). Additional information may also increase trust and system usability ratings. When no additional information was presented, we expected participants to make more accurate decisions when provided recommendations. However, the benefit of additional information on UV selection accuracy may be greater when not provided recommendations. With no recommendations, there could be costs to decision time or workload given increased processing demands to make unaided decisions, and these costs could be amplified when additional information is presented/increased. The absence of recommendations may also decrease trust and usability ratings.

**Table 1.** Summary of Predictions Regarding the Impact of Additional Information and Decision Recommendations on Outcome Measures.

Measure^a^	Impact of Additional Information	Impact of Decision Recommendation	Interaction Between Additional Information and Decision Recommendation
UV selection accuracy	Increased UV selection accuracy when additional information is present compared to absent. Increased UV selection accuracy with high compared to medium additional information.	Interpretation of a recommendation main effect would be qualified by the observation of the predicted interaction.	When not provided a recommendation, the benefit of the provision of additional information could be greater, compared to when a recommendation is provided.
Correct decision time	No difference in decision time.	Increased decision time when no recommendation is provided.	We tentatively predict that when not provided a recommendation, decision time may further increase when additional information is present.
Workload	No difference in workload.	Increased workload when no recommendation is provided.	We tentatively predict that when not provided a recommendation, workload may further increase when additional information is present.
Information accuracy discrimination	Improved information accuracy discrimination with high compared to medium additional information.	Improved information accuracy discrimination when no recommendation is provided.	No clear prediction.
Trust and system usability	Increased trust and system usability with high compared to medium additional information.	Decreased trust and system usability when no recommendation is provided.	No clear prediction.

*Note.* Additional information = absent (no), present (medium/high); medium, high. Recommendation = no recommendation provided, recommendation provided.

^a^Note that UV selection accuracy, correct decision time, and workload data was available for all conditions. Information accuracy discrimination data was only available for conditions with additional information to judge (i.e., medium and high). The no additional information condition was excluded from the trust and usability data because when no additional information and no recommendation were provided, it was not possible to collect this data as the task was completed manually.

Participants in the medium and high additional information conditions, regardless of whether recommendations were provided, also indicated whether the UV system had presented accurate versus inaccurate additional information on each trial (information accuracy discrimination). Despite inputting information accuracy responses after UV selection, participants likely verified additional information during the UV selection process, providing an indication of the extent to which additional information was scrutinized. We expected that information accuracy discrimination would improve when no recommendation was provided, indicative that participants hold higher expected value for processing additional information when not provided recommendations. We also expected improved information accuracy discrimination with the provision of high compared to medium additional information.

## Method

### Participants

One hundred and forty-two students (85 female, 55 male, 2 nonbinary; *M* = 20.2 years) at The University of Western Australia (UWA) participated for course credit and a performance incentive (max AUD$18). Participants were randomly assigned to either no (*n* = 46), medium (*n* = 48), or high (*n* = 48) additional information conditions. This research complied with American Psychological Association Code of Ethics and was approved by the UWA Human Research Ethics Office. Informed consent was obtained.

### Uninhabited Vehicle Management Task

The UV task was presented on a single desktop monitor. Participants completed 120 mission trials, split into two blocks of 60 trials (one block with recommendations provided, one block without recommendations provided).

#### Mission Trial

**Figure 1.**
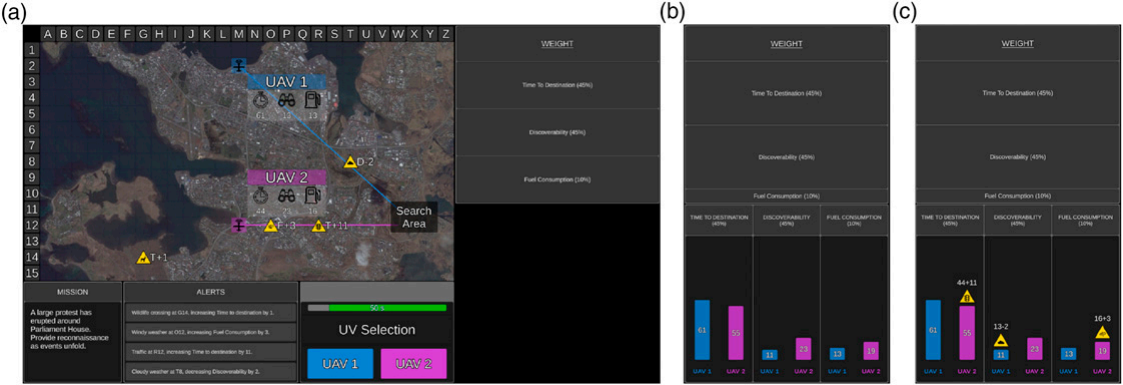
Example of (a) no, (b) medium, and (c) high additional information conditions when no recommendation was provided. The tactical map presented the two UVs (UAV1 and UAV2), their capabilities (time to destination, discoverability, fuel consumption) in a translucent grey box, the path each UV took to the search area (translucent black box), and environmental factors as factor symbols (three relevant and one irrelevant in this example). The UV Selection Window presented UAV1 on the left and UAV2 on the right, and the remaining time available for the mission. The weighting display presented each UV capability weighting as percentages (time to destination = 45%, discoverability = 45%, fuel consumption = 10%). Additional information was presented in the weighting and graph displays for medium and high additional information conditions. The weighting display showed the importance of UV capability weightings, with higher weightings depicted by larger sized rows (identical for (b) medium and (c) high additional information conditions; time to destination and discoverability were equally weighted in this example). For (b) medium additional information, the graph display showed a visual comparison of the calculated score for each UV capability, after taking relevant factors into account. For (c) high additional information, the graph display additionally showed *how* each UV capability score was calculated. Specifically, the environmental factor symbol was presented above the bar when the UV system considered a relevant factor, and if so, the original capability score (identical to the tactical map) plus/minus the numerical value of the environmental factor. The urban terrain map was used for this mission. *Note.* The Tactical Map and Mission, Alerts, and UV Selection Windows were identical for all conditions.

Participants were asked to select the optimal UV to complete each mission (trial). Mission statements were presented in the Mission Window for face validity but were not relevant to UV selection. The tactical map presented rural, urban, or coastal terrains, the search area, and two UVs. UVs were randomly numbered 1 or 2 and were aerial (UAV), ground (UGV), or surface (USV) vehicles. Accompanying each UV was a line indicating the path it would take to the search area, and the UVs’ capabilities (Figure 1).

UV capabilities included: time to destination (time required to reach search area), discoverability (how discoverable the UV was by third parties), and fuel consumption (fuel consumed to reach search area). On the tactical map, time to destination was depicted by a value next to a timer symbol (lower = quicker), discoverability by a binocular symbol (lower = less discoverable), and fuel consumption by a fuel gauge symbol (lower = less fuel). For each mission, UV capabilities had different weightings, presented as percentages in the weightings display, with higher weightings indicating greater mission relevance. Five weighting combinations were used; 60% of missions were assigned “harder” combinations and 40% assigned “easier” combinations. Harder combinations (e.g., 40%, 30%, and 30%) had two capabilities with equal weightings, and participants considered which UV scored lower on two capabilities. Easier combinations (e.g., 80%, 10%, and 10%) required participants to only consider which UV scored lower on one highly weighted capability.

During each mission, four environmental factors (e.g., traffic) were presented on the tactical map as yellow factor symbols. Next to each symbol was a letter (T = time to destination, D = discoverability, F = fuel consumption) representing the UV capability impacted, and a numerical value indicating the direction (positive/negative) and magnitude of impact. Relevant factors impacted UV capabilities and were depicted by symbols on a UV path, whereas irrelevant factors were not on a UV path. Each mission had between zero (i.e., all irrelevant) and three relevant factors. All four environmental factors and their associated impact were also presented in text form in the Alerts Window.

Based on UV capabilities, mission-specific weightings, and relevant environmental factors, participants selected the optimal UV to complete each mission. UV1 (colored blue) was presented on the left and UV2 (colored purple) on the right. Participants had 60s to make a decision. After each mission, feedback regarding UV selection accuracy and decision time was presented.

#### Additional Information

In the no additional information condition, no further display information was presented (Figure 1(a)). In the medium (Figure 1(b)) and high additional information (Figure 1(c)) conditions, information was displayed in the weighting and graph displays. The weighting display was identical for both conditions and showed how the UV system evaluated UV capability weightings. Specifically, lower weightings were depicted by smaller sized rows, and higher weightings by larger sized rows.

With medium additional information, the graph display had three bar graphs, one for each UV capability. The bar graphs showed the final calculated score (i.e., reasoning process) for each capability after considering relevant environmental factors (Figure 1(b)). Shorter bars indicated lower UV capability scores (i.e., better capability). Therefore, medium additional information, when recommendations were provided, broadly reflected SAT Level 2 transparency, by providing the final outcomes of the reasoning behind automated advice.

With high additional information, the graph also provided information regarding *how* the UV system had calculated the projected impact/consequences of relevant environmental factors for UV capability scores (Figure 1(c)). For each relevant environmental factor, the factor symbol was presented above the associated bar, along with the original capability score plus/minus the numerical value of the environmental factor. High additional information therefore outlined the UV system’s projected impact of environmental factors, and the projected outcomes of UV selection. Therefore, high additional information, when recommendations were provided, broadly reflected SAT Level 3 transparency, by providing the automation’s calculation and projection of the consequences of variability in the task environment, and thus projected outcomes if advice was actioned.

The UV system made calculation errors (provided inaccurate additional information) by either missing or miscalculating the magnitude of impact of a relevant factor. When there were multiple relevant factors, inaccurate information could be provided on one or more of these factors.

With medium additional information, if the UV system missed or miscalculated the impact of a relevant factor, the graph display would present an inaccurate UV capability score. With high additional information, if the UV system missed a relevant factor, the factor symbol would be missing in the graph display to show that it did not consider the factor in its calculation. Additionally, the original capability score plus/minus the magnitude of impact of the factor would be missing, and an inaccurate UV capability score was presented. If the UV system identified but miscalculated the impact of a relevant factor, the factor symbol was presented on the graph display to indicate that it had been considered. However, the value added/subtracted from the original capability score would be incorrect, resulting in an inaccurate UV capability score. When coupled with recommendations, high additional information was designed to increase automated advice understandability and projected outcomes if advice was followed (i.e., selecting the recommended UV). When not coupled with recommendations, the same information could be used to make unaided UV selections.

#### Decision Recommendation

All participants completed two 60-trial blocks (A and B)—one without recommendations and one with recommendations.

In the No Recommendation block, participants selected a UV from the UV Selection Window to complete each mission (Figure 1). In the medium and high additional information conditions, participants were instructed that the UV system was highly reliable, but not perfect, and it may not provide accurate additional information all the time.

**Figure 2.**
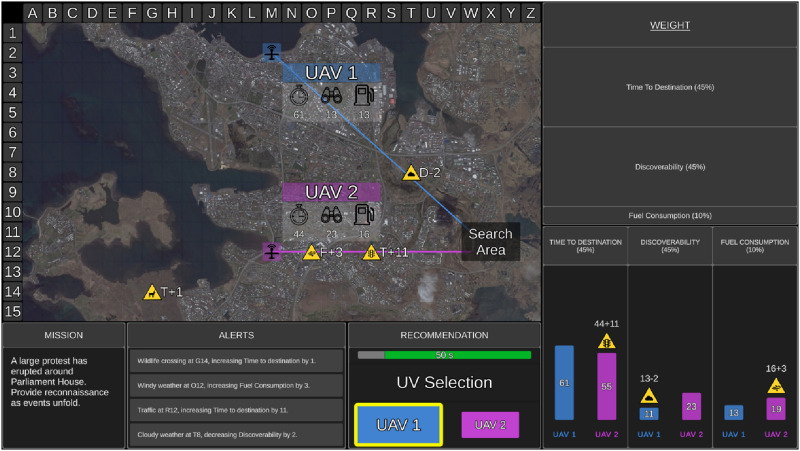
Example of a mission with a recommendation provided in the high additional information condition. The UV system presented its advice in the UV Selection (Recommendation) Window. The advised UV (UAV1) was outlined in yellow and larger than the alternative UV (UAV2). In this example, the UV system recommended the correct UV. For each mission, the UV system could have recommended UV1 or UV2 as the optimal UV to complete each mission.

In the Recommendation block, the UV system advised the optimal UV to complete missions based on UV capabilities, capability weightings, and the impact of relevant environmental factors. In the UV Selection (Recommendation) Window, the recommended UV was outlined in yellow, presented larger than the alternative UV, and could be either UV1 or UV2 (Figure 2). Participants were required to either choose the recommended UV or reject that advice and choose the alternative UV. While participants were not told the exact reliability, they were instructed that the UV system was highly reliable, but not perfect, and it may not always recommend the optimal UV and/or provide accurate additional information.

Participants in the medium and high additional information conditions (in both the recommendation and no recommendation blocks) were also required to determine whether the UV system provided accurate information on each mission (i.e., had correctly identified and calculated the impact of relevant environmental factor/s). After selecting a UV, a black screen appeared with two selection buttons. Participants had 3s to select whether the additional information was “Accurate” or “Inaccurate.” After selecting a UV in the no additional information condition, participants were presented a black display (instead of the information accuracy selection display) and a 3s countdown.

In addition to UV selection accuracy and decision time feedback presented to all conditions, the medium and high additional information conditions were also provided feedback regarding (a) whether the UV system provided accurate information and (b) whether the participant correctly discriminated if the information was accurate. During the Recommendation block only, feedback was also provided regarding whether the UV system had recommended the correct UV.

Table 2 shows the percentage of trials that the UV system recommended the correct UV and provided accurate/inaccurate information. The correct UV was recommended on 82.5% of trials, and the incorrect UV on 17.5% of trials. On these 17.5% of trials, the UV system calculation error/s were significant enough in direction and magnitude to result in the incorrect UV being recommended. In those instances, participants would be correct to choose the alternative UV, and to then select the “Inaccurate” button (medium/high additional information conditions only). For the same trials in no recommendation blocks, after selecting a UV, participants provided medium/high additional information would be correct to select the “Inaccurate” button.

**Table 2.** Percentage of Trials Where the UV System Recommended the Correct UV (Recommendation Block Only), and Percentage of Trials in Which the UV System Provided Accurate/Inaccurate Information for Medium and High Additional Information Conditions (in Both the Recommendation and No Recommendation Blocks).

Correct Recommended UV	Information Accurate/Inaccurate	Trials (%)
Yes	Accurate	65
Yes	Inaccurate	17.5
No	Inaccurate	17.5

Furthermore, in recommendation blocks of trials, on 17.5% of missions in which the correct UV was recommended, the UV system made inconsequential error/s in its calculations and thus provided inaccurate information. On these trials however, calculation error/s were not significant enough in direction/magnitude to result in the incorrect UV being recommended. These trials were included to minimize participants selecting the alternative UV when they detected any type of information error made by the UV system in the graph display (Gegoff et al., 2024; Tatasciore & Loft, 2024). In those instances, participants were instructed to select the recommended UV, but to then select the “Inaccurate” button. For the same 17.5% of trials in no recommendation blocks, after selecting a UV, participants provided medium/high additional information were instructed to select the “Inaccurate” button.

To equate mission difficulty across blocks A and B, there was a similar number of capability weighting combinations, relevant environmental factors (between 0 and 3), and number/type of calculation error/s (i.e., missing and/or miscalculating). Presentation order of block A and B was counterbalanced, as well as whether recommendations were presented in the first or second block. A randomized yoked design was used such that one participant from each additional information condition received the same randomized trial order within each block.

### Measures

#### UV Selection Accuracy and Decision Time

UV selection accuracy was the proportion of missions in which participants selected the correct UV. Decision time was calculated for correct decisions.

#### Workload

Assessed using the NASA Task Load Index (Hart & Staveland, 1988). Workload scores ranged from 0 (very low) to 100 (very high).

#### Information Accuracy Discrimination

The proportion of missions on which participants correctly identified whether additional information provided was accurate.

#### Trust

Assessed using an adapted version of the Merritt (2011) scale. The scale included six items rated on a 5-point Likert scale from 1 (strongly disagree) to 5 (strongly agree).

#### System Usability

Assessed using a modified version of the System Usability Scale (SUS; Brooke, 1996). This included 10 items measured on a 5-point Likert scale from 1 (strongly disagree) to 5 (strongly agree). Even items were reversed scored, and all items were added together and then multiplied by 2.5, resulting in final scores ranging from 0–100.

#### Attention Control

Participants’ attention control capacity was measured using the Three-Squared Task (Burgoyne et al., 2023); however, these data are not presented here.

### Procedure

Participants first completed 35 min of training, completing manual training without additional information or recommendations (15 min audio-visual presentation and 20 missions). This was followed by additional information condition-specific training. Participants then watched a block-specific (no recommendation or recommendation) training presentation and completed 60 missions in the assigned block. After a self-paced break of at least 60 s, participants watched a training presentation relevant to the second block of trials and completed 60 missions in that block. Questionnaires were administered after each block (counterbalanced and yoked order across conditions). The experiment lasted 2.5 hrs. To combat potential fatigue effects, participants were encouraged to take breaks before/after training presentations, blocks of trials, and questionnaire administration.

## Results

Data was excluded from two careless responders in the no additional information condition (responded to >3% of trials in <1 s) and one participant from the medium additional information condition who did not follow task instructions.

### Manual Training UV Selection Accuracy

One-way ANOVAs on manual training UV selection accuracy (*M* = .86, *SD* = .11) and correct decision time (*M* = 17.5 s, *SD* = 6.07 s) indicated no significant differences among the additional information conditions (smallest *p* = .34).

### Data Analysis and Statistics

Table 3 presents descriptive statistics for each dependent measure as a function of additional information and recommendation.

**Table 3.** Means (Standard Deviations) for UV Selection Accuracy, Correct Decision Time, Workload, Information Accuracy Discrimination, Trust, and System Usability Split by Additional Information and Recommendation.

	No Recommendation			Recommendation		
	No	Medium	High	No	Medium	High
UV selection accuracy	.84 (.13)	.87 (.12)	.85 (.13)	.86 (.08)	.92 (.08)	.89 (.09)
Correct decision time (s)	14.2 (5.37)	20.7 (5.02)	17.0 (6.67)	14.2 (5.21)	19.3 (5.08)	14.7 (6.62)
Workload	50.5 (16.5)	59.8 (14.4)	54.4 (14.1)	52.2 (13.6)	58.8 (14.3)	51.4 (16.4)
Information accuracy discrimination	-	.93 (.07)	.95 (.05)	-	.92 (.07)	.94 (.06)
Trust	-	2.10 (0.71)	2.51 (0.83)	2.62 (0.75)	2.18 (0.78)	2.62 (0.75)
System usability	-	56.6 (14.7)	60.4 (14.6)	66.7 (13.3)	58.3 (13.1)	64.7 (12.0)

*Note.* No = no additional information (i.e., absent), Medium = medium additional information, High = high additional information. Dashed lines represent data cells that were not possible to compute due to no measurement.

First, we ran 3 Additional Information (no, medium, high) × 2 Recommendation (no recommendation provided, recommendation provided) mixed ANOVAs to analyze UV selection accuracy, correct decision time, and workload, as these outcome variables were relevant to all conditions. The between-subjects factor was additional information, and the within-subjects factor was recommendation.

The importance of establishing the robustness and generality of psychological effects is well recognized (Pashler & Wagenmakers, 2012; Yong, 2012), including in the human factors literature (Jones et al., 2010). On this basis, regardless of whether interactions between additional information and recommendation were found, we made the a priori decision to conduct planned comparisons (Rosenthal & Rosnow, 1985) to allow potential replication of outcomes from our prior studies that presented additional information with recommendations (i.e., transparency) using the same or a highly similar UV task (Gegoff et al., 2024; Tatasciore et al., 2023, 2024; Tatasciore & Loft, 2024). Specifically, we report independent-samples *t*-tests comparing the impact of providing no (i.e., absent) versus medium/high (i.e., present) additional information, and the impact of the level of additional information presented (i.e., medium vs. high), at each level of the recommendation condition. To allow further comparison with our prior work, we report one-way ANOVAs using Signal Detection outcome measures that assess the impact of additional information on the accuracy of automation use in recommendation blocks (i.e., hit rate, correct rejection rate, sensitivity, and response bias) in Supplemental Materials 1.

Effect sizes for *F*-tests were estimated using partial eta squared ( $\eta_{\rho }^{2}$ ; small = .01, medium = .06, large = .14), and *t*-tests using Cohen’s *d* (small = .20, medium = .50, large = .80; Cohen, 1992).

We also tested for order effects (recommendation vs. no recommendation provided first) by conducting 3 Additional Information (no, medium, high) × 2 Recommendation (no recommendation provided, recommendation provided) × 2 Order (first, second) mixed ANOVAs. These analyses revealed some significant order effects but identified no statistical patterns that meaningfully changed our interpretation of the data, and for brevity, are presented in Supplemental Materials 2.

### UV Selection Accuracy

There was a main effect of recommendation, *F* (1,139) = 27.0, *p* < .001, $\eta_{\rho }^{2}$ = .16, with increased UV selection accuracy when recommendations were provided (*M* = .89, *SD* = .09) compared to not provided (*M* = .85, *SD* = .12). There was no main effect of additional information, *F* (2,139) = 2.27, *p* = .11, and no interaction, *F* (2,139) = 1.30, *p* = .28.

With recommendations provided, UV selection accuracy was higher when additional information was present (*M* = .91, *SD* = .09) compared to absent, *t* (140) = 2.76, *p* = .01, *d* = .50, but there was no difference between medium and high additional information conditions, *t* (94) = 1.42, *p* = .16. When no recommendations were provided, there was no difference in UV selection accuracy when additional information was present (*M* = .86, *SD* = .12) compared to absent, *t* < 1, or between medium and high additional information conditions, *t* (94) = 1.06, *p* = .29.

### Correct Decision Time

There was a main effect of additional information, *F* (2,139) = 15.9, *p* < .001, $\eta_{\rho }^{2}$ = .19. Participants made slower correct decisions when additional information was present (*M* = 17.8 s, *SD* = 5.60 s) compared to absent (*M* = 14.2 s, *SD* = 4.95 s), *t* (140) = 3.77, *p* < .001, *d* = .68, but faster correct decisions with high (*M* = 15.7 s, *SD* = 6.18 s) compared to medium (*M* = 19.9 s, *SD* = 4.03 s) additional information, *t* (80.8) = 3.94, *p* < .001, *d* = .80. There was also a main effect of recommendation, *F* (1,139) = 8.56, *p* = .004, $\eta_{\rho }^{2}$ = .06, with faster correct decisions with recommendations provided (*M* = 16.1 s, *SD* = 6.09 s) compared to not provided (*M* = 17.4 s, *SD* = 6.29 s). There was no interaction, *F* (2,139) = 2.40, *p* = .09.

With recommendations provided, participants made slower correct decisions when additional information was present (*M* = 17.0 s, *SD* = 6.29 s) compared to absent, *t* (140) = 2.58, *p* = .01, *d* = .46, but faster correct decisions with high compared to medium additional information, *t* (94) = 3.74, *p* < .001, *d* = .76. When no recommendations were provided, participants made slower correct decisions when additional information was present (*M* = 18.9 s*, SD* = 6.17 s) compared to absent, *t* (140) = 4.36, *p* < .001, *d* = .78, but faster correct decisions with high compared to medium additional information, *t* (87.3) = 3.15, *p* = .002, *d* = .64.

### Workload

There was a main effect of additional information, *F* (2,139) = 4.71, *p* = .01, $\eta_{\rho }^{2}$ = .06. While there was no difference in workload when additional information was present (*M* = 56.1, *SD* = 13.7) compared to absent (*M* = 51.3, *SD* = 13.6), *t* (140) = 1.95, *p* = .05, workload ratings were lower with high (*M* = 52.9, *SD* = 13.6) compared to medium (*M* = 59.3, *SD* = 13.2) additional information, *t* (94) = 2.36, *p* = .02, *d* = .48. There was no main effect of recommendation, *F* < 1, and no interaction, *F* (2,139) = 1.57, *p* = .21.

With recommendations provided, there was no difference in workload when additional information was present (*M* = 55.1, *SD* = 15.7) compared to absent, *t* (140) = 1.10, *p* = .27, but workload was rated lower with high compared to medium additional information, *t* (94) = 2.38, *p* = .02, *d* = .49. When no recommendations were provided, workload ratings were higher when additional information was present (*M* = 57.1, *SD* = 14.4) compared to absent, *t* (140) = 2.44, *p* = .02, *d* = .44, but there was no difference between medium and high additional information, *t* (94) = 1.88, *p* = .06.

### Information Accuracy Discrimination, Trust, and Usability

We analyzed the impact of additional information and recommendations on information accuracy discrimination, trust, and usability using 2 Additional Information (medium, high) × 2 Recommendation (no recommendation provided, recommendation provided) mixed ANOVAs. We then analyzed the impact of medium compared to high additional information, at each level of recommendation, with planned *t*-tests (Rosenthal & Rosnow, 1985).

The no additional information condition was excluded since these data were not available when no recommendation was provided (dashed lines in Table 3). However, we report the impact of additional information (no, medium, high) when recommendations were provided on trust and usability in Supplemental Materials 1 to facilitate comparison with our prior work (e.g., Gegoff et al., 2024; Tatasciore & Loft, 2024).

To examine order effects, we conducted 2 Additional Information (medium, high) × 2 Recommendation (no recommendation provided, recommendation provided) × 2 Order (first, second) mixed ANOVAs. These analyses revealed some significant order effects, but identified no statistical patterns that meaningfully changed our interpretation of the data, and for brevity, are presented in Supplemental Materials 2.

#### Information Accuracy Discrimination

There was a main effect of recommendation, *F* (1,94) = 4.55, *p* = .04, $\eta_{\rho }^{2}$ = .05, with participants better able to discriminate whether information was accurate when recommendations were not provided (*M* = .94, *SD* = .06) compared to provided (*M* = .93, *SD* = .07). There was no main effect of additional information, *F* (1,94) = 1.53, *p* = .22, and no interaction, *F* < 1.

There was no difference in information accuracy discrimination between medium and high additional information conditions with recommendations provided, *t* (94) = 1.26, *p* = .21, or not provided, *t* < 1.

#### Trust

There was a main effect of additional information, *F* (1,94) = 8.84, *p* = .004, $\eta_{\rho }^{2}$ = .09, with higher trust ratings with high (*M* = 2.57, *SD* = 0.73) compared to medium (*M* = 2.14, *SD* = 0.67) additional information. There was no main effect of recommendation, *F* (1,94) = 1.84, *p* = .18, and no interaction, *F* < 1.

Trust ratings were higher with high compared to medium additional information both with recommendations provided, *t* (94) = 2.82, *p* = .01, *d* = .58, and not provided, *t* (94) = 2.59, *p* = .01, *d* = .53.

#### Usability

There was a main effect of additional information, *F* (1,94) = 4.57, *p* = .04, $\eta_{\rho }^{2}$ = .05, with higher usability ratings with high (*M* = 62.6, *SD* = 11.7) compared to medium (*M* = 57.4, *SD* = 11.8) additional information. There was also a main effect of recommendation, *F* (1,94) = 4.48, *p* = .04, $\eta_{\rho }^{2}$ = .05, with higher usability ratings with recommendations provided (*M* = 61.5, *SD* = 12.9) compared to not provided (*M* = 58.5, *SD* = 14.7). There was no interaction, *F* < 1.

Usability ratings were higher with high compared to medium additional information with recommendations provided, *t* (94) = 2.51, *p* = .01, *d* = .51, but there was no difference when no recommendations were provided, *t* (94) = 1.27, *p* = .21.

## Discussion

We examined the effect of providing additional information with and without decision recommendations on decision (UV selection) accuracy, correct decision time, workload, trust, and usability. The no additional information condition received no additional information regarding UV capability weightings or the projected impact of environmental factors. Medium additional information presented information about the UV system’s evaluation of UV capability weightings, and a visual comparison of final calculated UV scores for each capability. High additional information presented further information regarding *how* the UV system calculated each UV capability score based on which environmental factors were considered and their projected impact on UV capabilities. When coupled with recommendations, medium, and to a greater extent high, additional information was designed to increase automated advice understandability and projected outcomes if advice was followed (i.e., increased transparency). When not coupled with recommendations, the same additional information could be used to inform unaided UV selections. The study predictions and findings are summarized in Table 4.

**Table 4.** Summary of Predictions and Findings Regarding the Impact of Additional Information and Decision Recommendations on Outcome Measures.

Measure	Impact of Additional Information	Prediction Supported?	Impact of Decision Recommendation	Prediction Supported?	Interaction Between Additional Information and Decision Recommendation	Prediction Supported?
UV selection accuracy	Increased UV selection accuracy when additional information is present compared to absent. Increased UV selection accuracy with high compared to medium additional information.	Not supported.	Interpretation of a recommendation main effect would be qualified by the observation of the predicted interaction.	N/A (but recommendations improved accuracy of automation use).	When not provided a recommendation, the benefit of the provision of additional information could be greater, compared to when a recommendation is provided.	Not supported.
Correct decision time	No difference in decision time.	Not supported. Increased decision time when additional information was present compared to absent. Decreased decision time with high compared to medium additional information.	Increased decision time when no recommendation is provided.	**Supported.**	We tentatively predict that when not provided a recommendation, decision time may further increase when additional information is present.	Not supported.
Workload	No difference in workload.	**Partially supported**. No difference in workload when additional information was present compared to absent. Decreased workload with high compared to medium additional information.	Increased workload when no recommendation is provided.	Not supported.	We tentatively predict that when not provided a recommendation, workload may further increase when additional information is present.	Not supported.
Information accuracy discrimination	Improved information accuracy discrimination with high compared to medium additional information.	Not supported.	Improved information accuracy discrimination when no recommendation is provided.	**Supported.**	No clear prediction.	No interaction.
Trust and system usability	Increased trust and system usability with high compared to medium additional information.	**Supported.**	Decreased trust and system usability when no recommendation is provided.	Not supported (trust). **Supported** (usability).	No clear prediction.	No interaction.

*Note.* Additional information = absent (no), present (medium/high); medium, high. Recommendation = no recommendation provided, recommendation provided.

### Impact of Additional Information

There was no main effect of additional information on UV selection accuracy or information accuracy discrimination, the latter being inconsistent with outcomes reported by Gegoff et al. (2024). Participants made slower correct decisions when additional information was present compared to absent, indicative of increased processing demands placed on participants to analyze additional information. However, the data in Table 3 and accompanying inferential statistics indicate that the increased decision time with additional information was driven by medium (rather than high) additional information. In fact, high (compared to medium) additional information resulted in faster correct decisions and lower workload. This is likely because our high additional information design, despite presenting more information, allowed for efficient information processing by using simple graphical symbols (Chen et al., 2014; NASEM, 2022). In contrast, some other prior UV studies (e.g., Bhaskara et al., 2021; Stowers et al., 2020) have used complex visualizations/text-based explanations, potentially increasing the information burden placed on humans.

Participants also trusted high additional information more than they trusted medium additional information, and rated it as more usable. However, for the recommendation condition, we do not know the degree to which participants trusted the recommendations versus the additional information presented by the UV system. Bangor et al. (2009) concluded that SUS scores between 50 and 70 (the range obtained here) only aligned to an adjective-anchored Likert scale rating of ok-to-good (marginally acceptable), indicating room for improvement in our additional information designs.

### Impact of Decision Recommendations

Providing decision recommendations resulted in better performance (increased UV selection accuracy), faster correct decisions, and higher usability ratings, compared to when recommendations were not provided. There was no difference in workload or trust as a function of recommendation provision. Taken together, these findings are consistent with the notion that providing recommendations can enhance human decision making (Bartlett & McCarley, 2017, 2021; Wickens & Dixon, 2007). However, it is still possible that participants were inefficient in how they integrated information from raw task inputs with recommendations and thus they may have used automated advice nonoptimally (Boskemper et al., 2022; Tikhomirov et al., 2023; Wang et al., 2009).

### Additional Information and Decision Recommendations

Some theorists have argued that humans may not engage with or scrutinize additional information at the level required to optimize decisions when also provided decision recommendations (Endsley, 2017; Miller, 2023). We theorized then, that it may be beneficial for modern work systems to provide additional information without recommendations because it may increase decision-maker agency and increase the expected value of processing additional information to inform unaided decisions (Moray, 2003; Senders, 1983; Wickens et al., 2015), potentially enhancing situation awareness and decision making. Partly in line with this, we found that participants better discriminated whether additional information was accurate when no recommendations were provided, indicating a greater depth of additional information processing. The finding that without recommendations, participants reported higher workload when additional information was present compared to absent, further supports the premise that participants scrutinized additional information more when not provided recommendations. Future research could use eye-tracking or information masking/uncovering methodologies to more precisely examine the extent to which operators attend to additional information with and without decision recommendations.

However, we found no support for our core prediction that the benefit of additional information would be greater when no recommendations were provided, in that no significant interaction was found between additional information and recommendation on UV selection accuracy. Nevertheless, motivated by the importance of replicating psychological effects (Jones et al., 2010; Yong, 2012), we made the a priori decision to conduct planned comparisons (Rosenthal & Rosnow, 1985). These indicated that, in contrast to the direction of the predicted interaction, additional information (absent vs. present) only enhanced UV selection accuracy when recommendations *were* provided. That is, additional information that provides operators with the reasoning process underlying display calculations (medium additional information), and the projected impact/consequences of environmental factors (high additional information), was only beneficial when accompanied with decision recommendations.

Through these planned analyses then, we replicated the benefit of additional information (i.e., present vs. absent) found by our prior studies on UV selection accuracy when decision recommendations were provided (i.e., better automated advice use; Gegoff et al., 2024; Tatasciore & Loft, 2024; Tatasciore et al., 2023, 2024). Furthermore, the findings reported in Supplemental Materials replicated Signal Detection outcomes reported in our prior UV task work. Specifically, when additional information was present compared to absent, sensitivity increased (*d'* range: 1.97–2.53), with participants more biased towards agreeing with recommendations. Overall, our data indicate benefits of additional information on the accuracy of automated advice use (i.e., transparency), in line with findings from a variety of other work domains (Bhaskara et al., 2020; Sargent et al., 2023; Van de Merwe et al., 2022), as well as other UV studies not conducted in our research laboratory (e.g., Mercado et al., 2016; Stowers et al., 2020).

### Limitations and Conclusions

A limitation was the use of novice participants that inevitably differ from experts in motivation and cognitive skills. In addition, task requirements in UV operations are more complex (U.S. Air Force, 2015), which may impact the generalizability of the current findings (Rieth & Hagemann, 2022). Further, we provided participants with immediate feedback on their accuracy, decision time, and recommendation accuracy (in relevant conditions) after each trial. This feedback may have influenced task outcomes. Future research should examine whether the current findings replicate under conditions where immediate feedback is not provided, particularly as immediate feedback may be unavailable in UV operations and other work settings.

Our UV task (recommendation condition) represents situations in which operators respond to proposals (decision recommendations) provided by automation (Van de Merwe et al., 2022). In other work contexts, operators supervise automated actions to intervene if required (e.g., Guznov et al., 2020; Skraaning & Jamieson, 2021). In these situations, transparency designs may take different forms to enhance automation understandability and predictability (e.g., making automation mode transitions more transparent, following automation activity in real time, or providing verbal feedback of current automation state).

In conclusion, to our knowledge, this is the first study to have tested whether the benefits of additional information differ based on whether decision recommendations are provided. We found no support for this prediction. In contrast, the findings are potentially indicative that, while individuals may indeed scrutinize display information more when not provided recommendations, it may still prove advantageous to provide additional information *with* decision recommendations (i.e., transparency) to support decision making.

## Key Points

• Aimed to decompose transparency to isolate the effects of additional information and decision recommendations on decision accuracy, decision time, perceived workload, trust, and system usability, in a simulated uninhabited vehicle (UV) management task.
• When decision recommendations were provided, participants made more accurate and faster decisions, and rated the UV system as more usable.
• Individuals made faster decisions, reported lower workload and higher trust, and rated the UV system as more usable, when provided high compared to medium additional information.
• No support found for the core prediction that the benefit of additional information would be greater when no recommendations were provided.
• Results suggested that participants scrutinized additional information more when not provided decision recommendations. However, in contrast to the direction of the predicted interaction, additional information only improved decisions when accompanied by recommendations to support decisions.

## Supplemental Material

Supplemental Material - Deciphering Automation Transparency: Do the Benefits of Transparency Differ Based on Whether Decision Recommendations are Provided? Supplemental Material for Deciphering Automation Transparency: Do the Benefits of Transparency Differ Based on Whether Decision Recommendations are Provided? by Isabella Gegoff, Monica Tatasciore, Vanessa K. Bowden, and Shayne Loft in Human Factors.
