# Supplementary material for: Deciphering Automation Transparency: Do the Benefits of Transparency Differ Based on Whether Decision Recommendations Are Provided?
Source: Hum Factors. 2025 Feb 3;67(8):776–94. doi: 10.1177/00187208251318465 (PMC12231875; doi:10.1177/00187208251318465)
Supplement: Supplemental Material - Deciphering Automation Transparency: Do the Benefits of Transparency Differ Based on Whether Decision Recommendations are Provided? [file sj-pdf-1-hfs-10.1177_00187208251318465.pdf]

Supplementary Materials 1

Supplementary Materials 1 aimed to replicate our prior automation transparency research that used the same or a similar version of the uninhabited vehicle (UV) management task, presenting additional information with decision recommendations (i.e., transparency; Gegoff et al., 2024; Tatasciore & Loft, 2024; Tatasciore et al., 2023, 2024).

We analyzed the impact of additional information (no, medium, high) when recommendations were provided on the accuracy of automation use, perceived trust, and usability using one-way ANOVAs. We then conducted planned comparisons examining the impact of no (i.e., absent) compared to medium/high (i.e., present) additional information, followed by comparing medium to high additional information.

Supplementary Material 1a: Accuracy of Automation Use (Recommendation Condition)

Accuracy of automation use (hit rate, correct rejection rate, sensitivity, criterion) was analyzed using Signal Detection Theory (SDT) analyses, consistent with our prior research (Gegoff et al., 2024; Tatasciore & Loft, 2024; Tatasciore et al., 2023, 2024). Hit rate was defined as the proportion of missions that participants selected the correctly recommended UV. Correct rejection rate was the proportion of missions that participants correctly selected the alternative UV (when the recommended UV was incorrect). The SDT parameter *d'* assessed the sensitivity of participants to correctly discriminate between when the recommendation was correct or incorrect. The parameter *c* assessed whether participants were biased towards agreeing with recommendations (negative values=greater bias). To adjust for extreme hit or false alarm values (1 or 0), rates of 1 were replaced with (n–0.5)/n, and rates of 0 were replaced with 0.5/n, where n is the number of signal (recommended UV correct) or noise (alternative UV correct) trials (Macmillan & Kaplan, 1985).

Table S1 presents descriptive statistics for accuracy of automation use, trust, and usability as a function of additional information (recommendation condition only).

Table S1
Means (standard deviations) for accuracy of automation use (hit, correct rejection, sensitivity, criterion), trust, and usability as a function of additional information (recommendation condition only).

	No	Medium	High
Hit	.88 (.07)	.95 (.07)	.93 (.09)
Correct rejection	.77 (.18)	.78 (.20)	.73 (.21)
Sensitivity (*d'*)	1.97 (0.83)	2.53 (0.98)	2.26 (1.01)
Criterion (*c*)	-.24 (.22)	-.46 (.28)	-.49 (.36)
Trust	2.62 (0.75)	2.18 (0.78)	2.62 (0.75)
Usability	66.7 (13.3)	58.3 (13.1)	64.7 (12.0)

Note. No = no additional information (i.e., absent), Medium = medium additional information, High = high additional information.

Hit rate. There was a significant difference in hit rate between additional information conditions, $F(2,139)=8.71$, $p<.001$, $\eta_p^2=.11$. Hit rates were higher when additional information was present ($M=.94$, $SD=.08$) compared to absent, $t(140)=3.99$, $p<.001$, $d=.72$. However, there was no difference in hit rates between medium and high additional information conditions, $t(94)=1.17$, $p=.24$.

Correct rejection rate. There was no difference in correct rejection rates between additional information conditions ($F<1$). There was no difference in correct rejection rates when additional information was present ($M=.76$, $SD=.21$) compared to absent, $t(140)=.29$, $p=.77$, or between medium and high additional information conditions, $t(94)=1.30$, $p=.20$.

Sensitivity. There was a significant difference in sensitivity (*d'*) between additional information conditions, $F(2,139)=4.12$, $p=.02$, $\eta_p^2=.06$. Participants were better able to discriminate between correct and incorrect recommendations when additional information was present ($M=2.39$, $SD=1.00$) compared to absent, $t(140)=2.48$, $p=.01$, $d=.45$, but there was no difference between medium and high additional information conditions, $t(94)=1.35$, $p=.18$.

Criterion. There was a significant difference in response bias (c) between additional information conditions, $F(2,139)=10.38$, $p<.001$, $\eta_p^2=.13$. Participants were more biased towards agreeing with recommendations when additional information was present ($M=-.48$, $SD=.32$) compared to absent, $t(124.6)=5.18$, $p<.001$, $d=.81$. There was no difference in response bias between medium and high additional information conditions ($t<1$).

Summary: The finding of increased hit rate, sensitivity, and response bias when additional information was present compared to absent is consistent with our prior work using the same or similar UV task paradigm (Gegoff et al., 2024; Tatasciore & Loft, 2024; Tatasciore et al., 2023, 2024). However, a notable distinction is the lack of difference in hit rate between medium and high additional information conditions reported in prior work (Gegoff et al., 2024; Tatasciore et al., 2023). In fact, we found the opposite (at least numerically), with higher hit rates with medium compared to high additional information. Nonetheless, the findings of no significant difference in correct rejection rate, sensitivity, or response bias between medium and high additional information conditions are consistent with prior work (Gegoff et al., 2024; Tatasciore et al., 2023).

Supplementary Material 1b: Trust and System Usability (Recommendation Condition)

Trust. There was a significant difference in trust ratings between additional information conditions, $F(2,139)=5.41$, $p=.01$, $\eta_p^2=.07$. There was no difference in trust when additional information was present ($M=2.40$, $SD=.79$) compared to absent, $t(140)=1.62$, $p=.11$. However, trust was higher with high compared to medium additional information, $t(94)=2.82$, $p=.01$, $d=.58$, an effect we have not previously found (Gegoff et al., 2024; Tatasciore et al., 2023).

Usability. There was a significant difference in perceived usability between additional information conditions, $F(2,139)=5.58$, $p=.01$, $\eta_p^2=.07$. Usability was rated lower when additional information was present ($M=61.5$, $SD=12.9$) compared to absent, $t(140)=2.12$, $p=.03$, $d=.40$. However, usability was rated higher with high compared to medium additional information, $t(94)=2.51$, $p=.01$, $d=.51$. This finding of higher usability with high compared to medium additional information is consistent with Gegoff et al. (2024), but not with Tatasciore et al. (2023).

Supplementary Materials 2

In this section, the main analyses from the paper are repeated with the inclusion of presentation order (recommendation vs no recommendation block presented first) as a factor. We analyze the impact of additional information, recommendation, and order on UV selection accuracy, correct decision time, and workload using 3 Additional Information (no, medium, high)×2 Recommendation (no recommendation provided, recommendation provided)×2 Order (first, second) mixed ANOVAs. Table S2 presents descriptive statistics for these dependent measures as a function of recommendation and order.

To follow-up significant interactions between recommendation and order, we conducted independent-samples *t*-tests examining whether recommendation presentation order resulted in differences in relevant outcome measures, at each level of recommendation. We also followed up main effects of additional information, or significant three-way interactions, with independent-samples *t*-tests comparing no (i.e., absent) to medium/high (i.e., present) additional information, followed by comparing medium to high additional information, at each level of recommendation and order where relevant.

Table S2

Means (standard deviations) for UV selection accuracy, correct decision time (in seconds), and workload as a function of recommendation (no recommendation, recommendation) and presentation order (first, second).

	No Recommendation		Recommendation	
	No Recommendation First	No Recommendation Second	Recommendation First	Recommendation Second
UV Selection accuracy	.84 (.13)	.86 (.12)	.88 (.10)	.90 (.07)
Correct decision time	19.1 (6.71)	15.6 (5.34)	17.8 (5.70)	14.4 (6.04)
Workload	52.6 (14.6)	57.3 (15.9)	54.6 (13.5)	53.7 (16.6)

UV Selection Accuracy

There was a main effect of recommendation, $F(1,136)=27.8$, $p<.001$, $\eta_p^2=.17$, with increased UV selection accuracy with recommendations provided (*M*=.89, *SD*=.09) compared to not provided (*M*=.85, *SD*=.12). There were no main effects of additional information,

$F(2,136)=2.23, p=.11$, or order $F<1$. There was an interaction between recommendation and order, $F(1,136)=7.13, p=.01, \eta_p^2=.05$.

Numerically, as indicated in Table S2, participants made more accurate UV selections in the second block compared to first block of trials completed, both when recommendations were provided and not provided. However, *t*-tests revealed that with recommendations provided, there was no difference in UV selection accuracy when recommendations were presented first or second, $t(128.9)=1.13, p=.26$. Similarly, when no recommendations were provided, there was no difference in UV selection accuracy when no recommendations were presented first or second, $t(140)=1.03, p=.30$. There was no interaction between additional information and recommendation, $F(2,136)=1.34, p=.27$, and no three-way interaction, $F<1$.

Correct Decision Time

There was a main effect of additional information, $F(2,136)=15.9, p<.001, \eta_p^2=.19$. Participants made slower correct decisions when additional information was present (M=17.8s, SD=5.60s) compared to absent (M=14.2s, SD=4.95s), $t(140)=3.77, p<.001, d=.68$, but faster correct decisions with high (M=15.7s, SD=6.18s) compared to medium (M=19.9s, SD=4.03s) additional information, $t(80.8)=3.94, p<.001, d=.80$. There was also a main effect of recommendation, $F(1,136)=17.4, p<.001, \eta_p^2=.11$, with faster correct decisions with recommendations provided (M=16.1s, SD=6.09s) compared to not provided (M=17.4s, SD=6.29s). There was no main effect of order $F<1$.

There was an interaction between additional information and recommendation, $F(2,136)=4.88, p=.01, \eta_p^2=.07$, and an interaction between recommendation and order, $F(1,136)=132.7, p<.001, \eta_p^2=.49$. However, these interactions were qualified by a three-way interaction between additional information, recommendation, and order, $F(2,136)=5.86, p=.004, \eta_p^2=.08$. Table S3 presents descriptive statistics for correct decision time as a function of additional information, recommendation, and order.

Table S3
Means (standard deviations) for correct decision time (in seconds) as a function of additional information, recommendation (no recommendation, recommendation), and presentation order (first, second).

	No Recommendation		Recommendation	
	No Recommendation First	No Recommendation Second	Recommendation First	Recommendation Second
No	14.3 (5.14)	14.1 (5.70)	16.1 (5.52)	12.3 (4.17)
Medium	23.3 (4.11)	18.1 (4.51)	21.1 (5.00)	17.4 (4.57)
High	19.5 (7.28)	14.5 (4.99)	16.0 (5.26)	13.5 (7.65)

Note. No=no additional information (i.e., absent), Medium=medium additional information, High=high additional information.

With no additional information, participants made faster correct decisions when recommendations were presented second compared to first, $t(44)=2.65$, $p=.01$, $d=.78$. However, when no recommendations were provided, there was no difference in correct decision times when no recommendations were presented first or second, $t<1$.

With medium additional information, participants made faster correct decisions when recommendations were presented second compared to first, $t(46)=2.64$, $p=.01$, $d=.76$, and when no recommendations were presented second compared to first, $t(46)=4.17$, $p<.001$, $d=1.20$.

With high additional information, there was no difference in correct decision times when recommendations were presented first or second, $t(46)=1.35$, $p=.18$. However, when no recommendations were provided, participants made faster correct decisions when no recommendations were presented second compared to first, $t(46)=2.78$, $p=.01$, $d=.80$.

In summary, except for when no recommendations were provided with no additional information, and despite not reaching statistical significance when recommendations were provided with high additional information, the findings simply reflect that participants made faster correct decisions in the second block compared to the first block of trials completed.

Workload

There was a main effect of additional information, $F(2,136)=4.81$, $p=.01$, $\eta_p^2=.07$. There was no difference in perceived workload when additional information was present ($M=56.1$, $SD=13.7$) compared to absent ($M=51.3$, $SD=13.6$), $t(140)=1.95$, $p=.05$. However,

perceived workload was lower with high (*M*=52.9, *SD*=13.6) compared to medium (*M*=59.3, *SD*=13.2) additional information, *t*(94)=2.36, *p*=.02, *d*=.48. There were no main effects of recommendation, *F*<1, or order, *F*(1,136)=1.69, *p*=.20. There were no interactions between additional information and recommendation, *F*(2,136)=1.60, *p*=.21, recommendation and order, *F*(1,136)=3.10, *p*=.08, and no three-way interaction, *F*(2,136)=1.09, *p*=.34.

Information Accuracy Discrimination, Trust, and Usability

We analyzed the impact of additional information, recommendation, and order on information accuracy discrimination, trust, and usability using 2 Additional Information (medium, high)×2 Recommendation (no recommendation provided, recommendation provided)×2 Order (first, second) mixed ANOVAs. Table S4 presents descriptive statistics for the dependent measures as a function of recommendation and order. The no additional information condition was excluded from these analyses as this data was not available when no recommendations were provided. Main effects of additional information and order, and significant interactions between recommendation and order, were followed up with independent-samples *t*-tests.

Table S4
Means (standard deviations) for information accuracy discrimination, trust, and usability as a function of recommendation (no recommendation, recommendation) and presentation order (first, second).

	No Recommendation		Recommendation	
	No Recommendation First	No Recommendation Second	Recommendation First	Recommendation Second
Information accuracy discrimination	.95 (.06)	.93 (.07)	.92 (.07)	.93 (.06)
Trust	2.31 (.72)	2.31 (.87)	2.11 (.67)	2.68 (.81)
Usability	58.1 (15.9)	59.0 (13.5)	58.4 (11.0)	64.6 (14.1)

Information accuracy discrimination. There was a main effect of recommendation, *F*(1,92)=4.46, *p*=.04, η_p^2=.05, with participants better able to discriminate whether information was accurate when no recommendations were provided (*M*=.94, *SD*=.06) compared to when recommendations were provided (*M*=.93, *SD*=.07). There were no main effects of additional information (medium vs high), *F*(1,92)=1.53, *p*=.22, or order, *F*<1. There

were no interactions between additional information and recommendation, recommendation and order, and no three-way interaction, all *Fs*<1.

Trust. There was a main effect of additional information, $F(1,92)=9.06$, $p=.003$, $\eta_p^2=.09$, with higher trust with high ($M=2.57$, $SD=0.73$) compared to medium ($M=2.14$, $SD=0.67$) additional information, $t(94)=2.97$, $p=.004$, $d=.61$. There was also a main effect of order, $F(1,92)=4.21$, $p=.04$, $\eta_p^2=.04$, with higher trust when recommendations were presented second ($M=2.50$, $SD=.71$) compared to first ($M=2.21$, $SD=.73$), $t(94)=1.98$, $p=.05$, $d=.40$. There was no main effect of recommendation, $F(1,92)=2.28$, $p=.13$.

There was an interaction between recommendation and order, $F(1,92)=23.3$, $p<.001$, $\eta_p^2=.20$. When recommendations were provided, trust was higher when recommendations were presented second compared to first, $t(90.7)=3.78$, $p<.001$, $d=.77$. However, when no recommendations were provided, there was no difference in trust when no recommendations when presented first or second, $t<1$. There was no interaction between additional information and recommendation, $F<1$, and no three-way interaction, $F(1,92)=1.08$, $p=.30$.

Usability. There was a main effect of additional information, $F(1,92)=4.58$, $p=.04$, $\eta_p^2=.05$, with higher usability ratings with high ($M=62.6$, $SD=11.7$) compared to medium ($M=57.4$, $SD=11.8$) additional information, $t(94)=2.14$, $p=.04$, $d=.44$. There was also a main effect of recommendation, $F(1,92)=4.73$, $p=.03$, $\eta_p^2=.05$, with higher usability ratings when recommendations were provided ($M=61.5$, $SD=12.9$) compared to not provided ($M=58.5$, $SD=14.7$). There was no main effect of order, $F(1,92)=1.20$, $p=.28$.

There was an interaction between recommendation and order, $F(1,92)=6.51$, $p=.01$, $\eta_p^2=.07$. When recommendations were provided, usability ratings were higher when recommendations were presented second compared to first, $t(94)=2.38$, $p=.02$, $d=.49$. When no recommendations were provided, there was no difference in usability ratings when no

recommendations were presented first or second, $t<1$. There was no interaction between additional information and recommendation, and no three-way interaction, $Fs<1$.

Summary

Presenting recommendations in the second compared to first block of trials resulted in faster correct decisions (except for the high additional information condition) and higher trust and usability ratings. When no recommendations were presented in the second compared to first block of trials, correct decisions were faster (except for the no additional information condition), however, there was no difference in trust or usability ratings. Collectively, the findings presented in Supplementary Materials 2 are indicative of practice effects.

References

Gegoff, I., Tatasciore, M., Bowden, V., McCarley, J., & Loft, S. (2024). Transparent automated advice to mitigate the impact of variation in automation reliability. *Human Factors, 66*(8), 2008–2024. https://doi.org/10.1177/00187208231196738

Macmillan, N. A., & Kaplan, H. L. (1985). Detection theory analysis of group data: Estimating sensitivity from average hit and false-alarm rates. *Psychological Bulletin*, *98*(1), 185–199. https://doi.org/10.1037/0033-2909.98.1.185

Tatasciore, M., & Loft, S. (2024). Can increased automation transparency mitigate the effects of time pressure on automation use? *Applied Ergonomics*, *114*, 104142–104142. https://doi.org/10.1016/j.apergo.2023.104142

Tatasciore, M., Bowden, V., & Loft, S. (2023). Do concurrent task demands impact the benefit of automation transparency? *Applied Ergonomics*, *110*, 104022–104022. https://doi.org/10.1016/j.apergo.2023.104022

Tatasciore, T., Strickland, L., & Loft, S. (2024). Transparency improves the accuracy of automation use, but automation confidence information does not. *Cognitive Research: Principles and Implications, 9*. https://doi.org/10.1186/s41235-024-00599-x
